# Quantifying the transmission of antimicrobial resistance at the human and livestock interface with genomics

**DOI:** 10.1016/j.cmi.2020.09.019

**Published:** 2020-12

**Authors:** Bryan A. Wee, Dishon M. Muloi, Bram A.D. van Bunnik

**Affiliations:** 1) Usher Institute, University of Edinburgh, Edinburgh, United Kingdom; 2) Centre for Immunity, Infection & Evolution, School of Biological Sciences, University of Edinburgh, Edinburgh, United Kingdom; 3) International Livestock Research Institute, Nairobi, Kenya

**Keywords:** Dynamics, Genomics, Human-livestock, Interface, One health, Sequencing, Transmission

## Abstract

**Background:**

Livestock have been implicated as a reservoir for antimicrobial resistance (AMR) that can spread to humans. Close proximity and ecological interfaces involving livestock have been posited as risk factors for the transmission of AMR. In spite of this, there are sparse data and limited agreement on the transmission dynamics that occur.

**Objectives:**

To identify how genome sequencing approaches can be used to quantify the dynamics of AMR transmission at the human–livestock interface, and where current knowledge can be improved to better understand the impact of transmission on the spread of AMR.

**Sources:**

Key articles investigating various aspects of AMR transmission at the human–livestock interface are discussed, with a focus on *Escherichia coli*.

**Content:**

We recapitulate the current understanding of the transmission of AMR between humans and livestock based on current genomic and epidemiological approaches. We discuss how the use of well-designed, high-resolution genome sequencing studies can improve our understanding of the human–livestock interface.

**Implications:**

A better understanding of the human–livestock interface will aid in the development of evidence-based and effective One Health interventions that can ultimately reduce the burden of AMR in humans.

## Introduction

Globally, the consumption of antibiotics in livestock production is increasing and has already surpassed the total mass consumed by humans [1]. As a result, there have been calls to restrict antibiotic usage in livestock under the assumption that this will also reduce the incidence of antimicrobial-resistant (AMR) organisms in humans [[2], [3], [4]]. However, there is still limited quantitative evidence for the level of resistance transmission occurring between livestock and humans and, more importantly, how much of an impact this has on the incidence of resistant bacteria in humans.

Antimicrobials are used in livestock for a variety of reasons, including disease treatment, prophylaxis and growth promotion. Antimicrobial use for growth promotion, often involving sub-therapeutic doses, has been banned in European Union countries since 2005. As of 2019, 118 (*n* = 153; 77%) World Organisation for Animal Health (OIE) member countries have stopped using antimicrobials as growth promoters in animals [5].

The relationship between levels of AMR in livestock and the occurrence in humans is complex: there are numerous combinations of antimicrobials, bacterial strains, mobile genetic elements (MGEs) and livestock species, each with their own dynamics [6]. Robust quantitative evidence showing the major pathways of dissemination of AMR bacteria, or their resistance determinants from food animals to humans will be key to the development of effective policies on antimicrobial stewardship and infection control for both human and animal health.

The application of high-resolution genome sequencing has the potential to be a powerful tool to help with our baseline understanding of these pathways. However, these technologies need to be employed together with rigorous study design and correct interpretation. The current literature shows a wide range of perspectives on this topic, ranging from studies that fail to find any overlap between livestock and humans, to studies that show evidence of closely related resistance determinants shared between humans and livestock [[7], [8], [9]]. The aim of this review is to briefly describe the various aspects of the human–livestock interface as we currently understand it, and how we can best apply genomic sequencing technologies to further inform our understanding of the dynamics of AMR at this interface.

In this review, we focus on *Escherichia coli*, a priority pathogen because of its widespread levels of antimicrobial resistance. The monitoring of AMR in *E. coli* in livestock and food products as an indicator organism for AMR is also supported by its distribution in the gastrointestinal tract of humans and animal species. Furthermore, *E. coli* is also involved in the sharing of MGEs with other bacteria that are found in similar environmental niches [10].

## Defining the human and livestock interface

Overlapping ecologies between livestock and human populations create diverse interfaces, which present opportunities for either population to act as a reservoir from which antimicrobial-resistant bacteria (ARB) or their antimicrobial resistance genes (ARGs) could be transmitted in either direction [11]. There are two conceptual models for the transmission of AMR between humans and livestock; clonal transfer of ARB and horizontal transmission of ARGs [6]. Furthermore, transmission can be direct or indirect. Direct transmission implies contact between humans and livestock through close proximity, whereas indirect transmission involves an intermediate between the two populations. Indirect transmission can either involve an environmental component such as the soil, animal manure, sewage and surface water, or an intermediate vector such as wild animals, invertebrate vectors or food-borne infections [[12], [13], [14], [15]]. The distinction between direct or indirect transmission of AMR between human and livestock populations is invariably difficult to assess without clear evidence of an intermediate step.

Humans are constantly exposed to bacteria in the environment with some of these encounters resulting in transient colonization or infection [16], although quantitative human health risk assessments are lacking. In the case of *E. coli*, ingested bacteria are transported to the gastrointestinal tract where they have to compete against the existing microbiota as well as the immune system [17]. One factor that determines successful colonization is the physiological similarity between the livestock and human host. Upon colonization, depending on its pathogenic potential, the growth of ARB could lead to a symptomatic infection or persistence as a commensal. Both of these scenarios will give ARB the opportunity to spread to other humans or horizontally transfer ARGs to other bacteria [18,19].

The potential of ARB to spread can also be influenced by the behaviour of the human host and the environment that they are in. Socio-economic conditions can influence AMR transmission at the human–livestock interface. For example, domestic settings in low- and middle-income countries in which people live in close proximity to livestock are hypothesized to present ideal conditions for cross-species dissemination of resistant pathogens [20]. These practices are economically important and are likely to continue, so it is important to identify and mitigate high-risk practices that can promote transmission between humans and livestock in this setting.

Horizontal gene transfer of ARGs via MGEs such as plasmids, expands the human–livestock interface beyond the confines of clonal transmission [21]. The association of ARGs with promiscuous mobile elements and less fastidious bacterial hosts that can thrive in diverse environmental conditions increases the opportunities for spread between human and livestock populations [22]. Identical ARG sequences have been found across distantly related bacterial strains, and even across different species and genera [23]. Genomic investigations have also revealed highly related ARGs and mobile elements in bacterial lineages across humans and livestock [7].

## Using genomics to understand the importance of each component of the human–livestock interface

Each facet of the human–livestock interface will have varying levels of contribution to the transmission of resistance, and various studies have been carried out to understand the interaction between bacteria, MGEs, hosts and the environment [2]. Whole genome sequencing is a critical tool in our ability to infer transmission of ARB and ARGs between livestock and humans in conjunction with epidemiological evidence such as spatial and temporal connectivity.

One conspicuous example of ARB transmission occurs during food-borne contamination due to symptomatic infections in humans that sometimes require hospitalization. One well-studied pathogen is the ruminant-associated *E. coli* O157 lineage that causes outbreaks of acute gastroenteritis in humans [[24], [25], [26], [27]]. Epidemiological studies in the USA have shown that the majority (52%–65%) of O157 outbreaks were linked to contaminated animal and plant food products; however, only 3%–10% of outbreaks were due to animal contact [28,29]. Some of these cases were traced back to the abattoirs where the meat was processed. These studies also show that only about one-tenth (10%–14%) of *E. coli* O157 infections spread from person to person and this is supported by analyses of large genomic data sets, suggesting that a similar fraction of O157 genotypes have the potential to cause infections in humans [30].

A paradigm of extensive and sustained community transmission of resistant *E. coli* is the pandemic ST131 lineage, which has been spreading through human populations since the 1980s [31]. Phylogenetic analyses of whole genomes show that a ST131 sub-lineage (H22) of human and poultry origin are intermixed, suggesting repeated introductions from a poultry source [32]. However, the relatively large genetic and geographical distances between isolates of human and avian origin in ST131 present some uncertainty in establishing clear directionality of transmission and do not rule out the possibility of an intermediate reservoir.

The spread of ARGs via plasmids and other types of MGEs is even harder to quantify with sequencing because of their mosaic nature and diversity. Two extreme scenarios of plasmid spread have been observed. Globally dispersed plasmids found in multiple genera and across large geographical areas have been found [33]. Conversely, the extended spectrum β-lactamase *bla*_CTX-M-15_ gene present in a clonal lineage of *E. coli* ST131 is encoded by a diverse array of IncF plasmids [34]. Experimental studies have also shown that the transfer of ARG mediated by plasmids does occur between *E. coli* and other *Enterobacteriaceae* in the environment or in the host [7,[35], [36], [37]].

## Improving our understanding of the human–livestock interface with well-designed genomic studies

The use of high-resolution genomics to investigate genetic sharing of AMR between livestock and humans should be complemented by well-designed studies that are able to capture the variation that intrinsically exists in the populations of interest so that the results are a true reflection of what is happening. Sample sizes should also be large enough for sufficient statistical power and sensitivity. In the case of single-isolate whole genome sequencing, the number of isolates collected should reflect the diversity expected to be present in the sampling area. This can be in the hundreds for small and less diverse communities or in the thousands for larger or more diverse communities. A sample size calculation should be carried out while designing a new study taking into account the expected effect size, margin of error and confidence levels.

The culturing and sequencing of thousands of bacterial isolates can pose a logistical challenge and may not always be feasible. One alternative is an isolate-independent approach of using whole metagenome sequencing to identify any genetic material present in the sample [38]. However, this method can have a low sensitivity depending on the composition of the sample and sequencing depth used. One solution is to employ a targeted sequencing approach with specially designed probes to enrich for known ARGs [39,40]. The use of metagenomics also allows for the assessment of variation in the composition of bacteria and AMR across different host populations [41]. When employed together with innovative approaches such as metagenome Hi-C, which provides information on the spatial proximity of DNA molecules, metagenomics can also be used to determine the association between ARG and bacterial species [42].

The identification of closely related bacterial isolates based on whole genome sequence identity is used to infer whether a recent transmission event could have occurred between two sources of isolation. A genetic distance threshold is used to define when two isolates are epidemiologically related. This threshold, whether based on nucleotide polymorphisms or on differences in core gene profiles, is typically based on the diversity observed in earlier studies for a bacterial species [43]. Other types of supporting epidemiological and phylogenetic evidence, such as temporal and geospatial structuring of samples and DNA substitution rate of the strain, also need to be taken into account when making this inference [43,44]. Increased sampling of ARB will also give a clearer picture of the variation of a given bacterial community in an environmental niche [45]. Furthermore, single isolate sequencing can only identify a single genotype as it requires a single colony to be selected from a potentially heterogeneous sample, unless multiple colonies from the same sample are taken [46].

The inability to find epidemiologically related isolates does not rule out transmission. ARB may be part of a diverse population found within a host and may not be detected without sufficiently sensitive sampling [47]. ARB can also colonize transiently, being absent at the time of sampling, but present long enough to spread ARGs to other commensal bacteria [48]. Longitudinal studies of various species of ARB are required to understand their stability and transmission rates in human populations. Higher than expected sequence diversity can also arise as the result of homologous recombination events or increased mutation rates, leading to false negatives if an over-stringent definition of relatedness is used [49].

Genomic analyses can also be used to quantify the role of MGEs in the transmission of AMR between human and livestock populations. The existence of identical MGE sequences in different hosts is an indicator of recent ARG transmission between populations [7]. Nevertheless, there is limited understanding of the rate of mutation and reassortment of MGEs over time, making the estimation of divergence time difficult. A better understanding of MGE diversity is needed before clear conclusions can be made based on the (dis-)similarity of MGEs found between human and livestock populations. The use of long read sequencing technologies such as those developed by Oxford Nanopore and Pacific Biosciences can also help to resolve repeated DNA sequences that preclude the reconstruction of longer plasmid sequences with short read technologies [50,51]. Further, the success of ARG identification is hinged on the comprehensiveness and quality of the AMR gene databases used—most of which are heavily biased towards human pathogens, and commonly studied bacteria, so choosing appropriate databases is imperative [52].

The applicability of studies carried out in different locations around the world should also be carefully considered before extrapolating findings to other settings that have substantial differences in infrastructure and environmental conditions. For example, a global metagenomic survey of AMR prevalence in sewage showed clear differences in regional prevalence that were driven largely by the level of development [12].

Every effort should be made to select a representative sample to answer the question that is set out in the study design. In order to find evidence for sharing of AMR at the human–livestock interface, the human and livestock samples should be matched both spatially and temporally in the best possible way, i.e. they should be epidemiologically linked rather than based on convenience sampling of populations with no direct associations. This can be a problem because of culture biases where clinical isolates from symptomatic infections are selected for sequencing or simply the presence of sufficient numbers for successful culture. Future research will benefit from designing studies in which epidemiologically linked human and livestock populations are systematically sampled, preferably longitudinally [53]. Moreover, as there is considerable diversity within human populations (e.g. healthy individuals versus hospitalized patients) and livestock (e.g. free range versus intensive farming), the specific population under consideration might impact their exposure to diverse groups of bacteria. Future studies investigating transmission of AMR between humans and food animals should therefore clearly clarify the subpopulations studied, including clearly defining the control groups used. In addition, inclusion of detailed data on antimicrobial usage in these populations should be considered [8].

Phylogenetic methods, which reconstruct both phylogeny and demographic history, can also be used to estimate the rate of transmission between humans and livestock from genome sequences [54]. This approach requires a well-sampled lineage of bacteria that has diverged recently enough to obtain a robust phylogeny not significantly affected by homologous recombination. These methods can be sensitive to over-sampling and under-sampling of isolates from different host populations and different sampling times, as the result of a violation of the assumption in the models that the number of samples from each population reflects the proportion of colonized individuals in each population [55,56]. The demographic assumptions made by each phylogenetic model should be taken into account when selecting the samples for analysis [57].

Finally, it is of utmost importance that any metadata that goes with the samples are recorded accurately using harmonized protocols guided by good data management guidelines [58]. Metadata should at bare minimum include the date and place of the sample and include detailed denominator data about the origin of the samples [8]. Ideally, all these data should be made publicly available for repeatability and use in future studies.

## Conclusions

Ensuring that future generations have access to effective antimicrobials is high on the agenda for many countries. Failure to tackle AMR threatens the attainment of various Sustainable Development Goals, including those on poverty reduction, reduced inequalities, clean water and sanitation, and progress already made will be lost [59,60]. Concepts such as the One Health approach embody the idea of an increasingly connected world and emphasize the importance of controlling antimicrobial exposure in all microbial habitats—humans, animals and the environment alike. The advances and accessibility of genomic sequencing technologies and innovative analytical methods are essential in improving our understanding of AMR transmission dynamics at the human–livestock interface. Together with good experimental design, surveillance frameworks can be established to capture the transmission dynamics at more informative epidemiological scales as well as across ecological interfaces. This ensures that the key drivers of resistance transmission between humans and livestock can be accurately identified and the most appropriate interventions can be adopted.

## Transparency declaration

The authors declare that they have no conflicts of interest.

B.W. was funded through the project ‘Selection and Transmission of Antimicrobial Resistance in Complex Systems (STARCS)’ in the 10.13039/100013281Joint Programming Initiative on Antimicrobial Resistance, Grant Reference: MR/R000093/1. D.M. was supported by the Darwin Trust of Edinburgh and by the UK Medical Research Council, Biotechnology and Biological Science Research Council (UK), the Economic and Social Research Council (UK), the Natural Environment Research Council (UK), through the Environmental & Social Ecology of Human Infectious Diseases Initiative (ESEI), Grant Reference: G1100783/1. B.v.B. was funded by 10.13039/501100011747Novo Nordisk Foundation through the project ‘Global Surveillance of Antimicrobial Resistance’ (Grant Reference: NNF16 OC0021856) and through the project ‘Selection and Transmission of Antimicrobial Resistance in Complex Systems (STARCS)’ in the Joint Programming Initiative on Antimicrobial Resistance.

## Authors' contributions

All authors contributed to literature search, manuscript preparation, editing and revisions.

## References

[1] Boeckel T.P.V., Glennon E.E., Chen D., Gilbert M., Robinson T.P., Grenfell B.T. (2017). Reducing antimicrobial use in food animals. Science.

[2] Hoelzer K., Wong N., Thomas J., Talkington K., Jungman E., Coukell A. (2017). Antimicrobial drug use in food-producing animals and associated human health risks: what, and how strong, is the evidence?. BMC Vet Res.

[3] O’Neill J. (2015). Antimicrobials in agriculture and the environment: reducing unnecessary use and waste. https://amr-review.org/sites/default/files/Antimicrobials%20in%20agriculture%20and%20the%20environment%20-%20Reducing%20unnecessary%20use%20and%20waste.pdf.

[4] Tang K.L., Caffrey N.P., Nóbrega D.B., Cork S.C., Ronksley P.E., Barkema H.W. (2017). Restricting the use of antibiotics in food-producing animals and its associations with antibiotic resistance in food-producing animals and human beings: a systematic review and meta-analysis. Lancet Planet Health.

[5] OIE (2020). OIE annual report on antimicrobial agents intended for use in animals. Better understanding of the global situation.

[6] Chang Q., Wang W., Regev-Yochay G., Lipsitch M., Hanage W.P. (2015). Antibiotics in agriculture and the risk to human health: how worried should we be?. Evol Appl.

[7] de Been M., Lanza V.F., de Toro M., Scharringa J., Dohmen W., Du Y. (2014). Dissemination of cephalosporin resistance genes between *Escherichia coli* strains from farm animals and humans by specific plasmid lineages. PLoS Genet.

[8] Muloi D., Ward M.J., Pedersen A.B., Fevre E.M., Woolhouse M.E.J., van Bunnik B.A.D. (2018). Are food animals responsible for transfer of antimicrobial-resistant *Escherichia coli* or their resistance determinants to human populations? A systematic review. Foodborne Pathog Dis.

[9] Ludden C., Raven K.E., Jamrozy D., Gouliouris T., Blane B., Coll F. (2019). One Health genomic surveillance of *Escherichia coli* demonstrates distinct lineages and mobile genetic elements in isolates from humans versus livestock. MBio.

[10] European Food Safety Authority, European Centre for Disease Prevention and Control (2019). The European Union summary report on antimicrobial resistance in zoonotic and indicator bacteria from humans, animals and food in 2017. EFSA J.

[11] Hassell J.M., Begon M., Ward M.J., Fèvre E.M. (2017). Urbanization and disease emergence: dynamics at the wildlife–livestock–human interface. Trends Ecol Evol.

[12] Hendriksen R.S., Munk P., Njage P., van Bunnik B., McNally L., Lukjancenko O. (2019). Global monitoring of antimicrobial resistance based on metagenomics analyses of urban sewage. Nat Commun.

[13] Muloi D., Kiiru J., Ward M.J., Hassell J.M., Bettridge J.M., Robinson T.R. (2019). Epidemiology of antimicrobial-resistant *Escherichia coli* carriage in sympatric humans and livestock in a rapidly urbanizing city. Int J Antimicrob Agents.

[14] Hassell J.M., Ward M.J., Muloi D., Bettridge J.M., Robinson T.P., Kariuki S. (2019). Clinically relevant antimicrobial resistance at the wildlife–livestock–human interface in Nairobi: an epidemiological study. Lancet Planet Health.

[15] Onwugamba F.C., Fitzgerald J.R., Rochon K., Guardabassi L., Alabi A., Kühne S. (2018). The role of “filth flies” in the spread of antimicrobial resistance. Travel Med Infect Dis.

[16] Ashbolt N.J., Amézquita A., Backhaus T., Borriello P., Brandt K.K., Collignon P. (2013). Human Health Risk Assessment (HHRA) for environmental development and transfer of antibiotic resistance. Environ Health Perspect.

[17] Kamada N., Chen G.Y., Inohara N., Núñez G. (2013). Control of pathogens and pathobionts by the gut microbiota. Nat Immunol.

[18] Bosch T., Schade R., Landman F., Schouls L., van Dijk K. (2019). A blaVIM-1 positive *Aeromonas hydrophila* strain in a near-drowning patient: evidence for interspecies plasmid transfer within the patient. Future Microbiol.

[19] Huddleston J.R. (2014). Horizontal gene transfer in the human gastrointestinal tract: potential spread of antibiotic resistance genes. Infect Drug Resist.

[20] Rousham E.K., Unicomb L., Islam M.A. (2018). Human, animal and environmental contributors to antibiotic resistance in low-resource settings: integrating behavioural, epidemiological and One Health approaches. Proc R Soc B Biol Sci.

[21] Cury J., Oliveira P.H., de la Cruz F., Rocha E.P.C. (2018). Host range and genetic plasticity explain the coexistence of integrative and extrachromosomal mobile genetic elements. Mol Biol Evol.

[22] Wyres K.L., Holt K.E. (2018). *Klebsiella pneumoniae* as a key trafficker of drug resistance genes from environmental to clinically important bacteria. Curr Opin Microbiol.

[23] Hu Y., Yang X., Li J., Lv N., Liu F., Wu J. (2016). The bacterial mobile resistome transfer network connecting the animal and human microbiomes. Appl Environ Microbiol.

[24] Butcher H., Elson R., Chattaway M.A., Featherstone C.A., Willis C., Jorgensen F. (2016). Whole genome sequencing improved case ascertainment in an outbreak of Shiga toxin-producing *Escherichia coli* O157 associated with raw drinking milk. Epidemiol Infect.

[25] Dallman T.J., Byrne L., Ashton P.M., Cowley L.A., Perry N.T., Adak G. (2015). Whole-genome sequencing for national surveillance of Shiga toxin-producing *Escherichia coli* O157. Clin Infect Dis Off Publ Infect Dis Soc Am.

[26] Rowell S., King C., Jenkins C., Dallman T.J., Decraene V., Lamden K. (2016). An outbreak of Shiga toxin-producing *Escherichia coli* serogroup O157 linked to a lamb-feeding event. Epidemiol Infect.

[27] Chase-Topping M., Gally D., Low C., Matthews L., Woolhouse M. (2008). Super-shedding and the link between human infection and livestock carriage of Escherichia coli O157. Nat Rev Microbiol.

[28] Heiman K.E., Mody R.K., Johnson S.D., Griffin P.M., Gould L.H. (2015). *Escherichia coli* O157 outbreaks in the United States, 2003–2012. Emerg Infect Dis.

[29] Rangel J.M., Sparling P.H., Crowe C., Griffin P.M., Swerdlow D.L. (2005). Epidemiology of *Escherichia coli* O157:H7 outbreaks, United States, 1982-2002. Emerg Infect Dis.

[30] Lupolova N., Dallman T.J., Matthews L., Bono J.L., Gally D.L. (2016). Support vector machine applied to predict the zoonotic potential of *E. coli* O157 cattle isolates. Proc Natl Acad Sci USA.

[31] Zakour N.L.B., Alsheikh-Hussain A.S., Ashcroft M.M., Nhu N.T.K., Roberts L.W., Stanton-Cook M. (2016). Sequential acquisition of virulence and fluoroquinolone resistance has shaped the evolution of *Escherichia coli* ST131. MBio.

[32] Liu C.M., Stegger M., Aziz M., Johnson T.J., Waits K., Nordstrom L. (2018). *Escherichia coli* ST131-H22 as a foodborne uropathogen. MBio.

[33] Petersen J., Vollmers J., Ringel V., Brinkmann H., Ellebrandt-Sperling C., Spröer C. (2019). A marine plasmid hitchhiking vast phylogenetic and geographic distances. Proc Natl Acad Sci USA.

[34] Johnson T.J., Danzeisen J.L., Youmans B., Case K., Llop K., Munoz-Aguayo J. (2016). Separate F-type plasmids have shaped the evolution of the H30 subclone of *Escherichia coli* sequence type 131. MSphere.

[35] Blake D.P., Hillman K., Fenlon D.R., Low J.C. (2003). Transfer of antibiotic resistance between commensal and pathogenic members of the *Enterobacteriaceae* under ileal conditions. J Appl Microbiol.

[36] Evans D.R., Griffith M.P., Sundermann A.J., Shutt K.A., Saul M.I., Mustapha M.M. (2020). Systematic detection of horizontal gene transfer across genera among multidrug-resistant bacteria in a single hospital. ELife.

[37] Schjørring S., Struve C., Krogfelt K.A. (2008). Transfer of antimicrobial resistance plasmids from *Klebsiella pneumoniae* to *Escherichia coli* in the mouse intestine. J Antimicrob Chemother.

[38] Bragg L., Tyson G.W., Paulsen I.T., Holmes A.J. (2014). Metagenomics using next-generation sequencing. Environmental microbiology: methods and protocols.

[39] Lanza V.F., Baquero F., Martínez J.L., Ramos-Ruíz R., González-Zorn B., Andremont A. (2018). In-depth resistome analysis by targeted metagenomics. Microbiome.

[40] Guitor A.K., Raphenya A.R., Klunk J., Kuch M., Alcock B., Surette M.G. (2019). Capturing the resistome: a targeted capture method to reveal antibiotic resistance determinants in metagenomes. Antimicrob Agents Chemother.

[41] Pehrsson E.C., Tsukayama P., Patel S., Mejía-Bautista M., Sosa-Soto G., Navarrete K.M. (2016). Interconnected microbiomes and resistomes in low-income human habitats. Nature.

[42] Stalder T., Press M.O., Sullivan S., Liachko I., Top E.M. (2019). Linking the resistome and plasmidome to the microbiome. ISME J.

[43] Schürch A.C., Arredondo-Alonso S., Willems R.J.L., Goering R.V. (2018). Whole genome sequencing options for bacterial strain typing and epidemiologic analysis based on single nucleotide polymorphism versus gene-by-gene–based approaches. Clin Microbiol Infect.

[44] Stimson J., Gardy J., Mathema B., Crudu V., Cohen T., Colijn C. (2019). Beyond the SNP threshold: identifying outbreak clusters using inferred transmissions. Mol Biol Evol.

[45] Paterson G.K., Harrison E.M., Murray G.G.R., Welch J.J., Warland J.H., Holden M.T.G. (2015). Capturing the cloud of diversity reveals complexity and heterogeneity of MRSA carriage, infection and transmission. Nat Commun.

[46] Köser C.U., Fraser L.J., Ioannou A., Becq J., Ellington M.J., Holden M.T.G. (2014). Rapid single-colony whole-genome sequencing of bacterial pathogens. J Antimicrob Chemother.

[47] Reeves P.R., Liu B., Zhou Z., Li D., Guo D., Ren Y. (2011). Rates of mutation and host transmission for an *Escherichia coli* clone over 3 years. PloS One.

[48] Kantele A., Kuenzli E., Dunn S.J., Dance D.A., Newton P.N., Davong V. (2020). Real-time sampling of travelers shows intestinal colonization by multidrug-resistant bacteria to be a dynamic process with multiple transient acquisitions. BioRxiv.

[49] Sánchez-Busó L., Comas I., Jorques G., González-Candelas F. (2014). Recombination drives genome evolution in outbreak-related *Legionella pneumophila* isolates. Nat Genet.

[50] Lemon J.K., Khil P.P., Frank K.M., Dekker J.P. (2017). Rapid nanopore sequencing of plasmids and resistance gene detection in clinical isolates. J Clin Microbiol.

[51] Arredondo-Alonso S., Willems R.J., van Schaik W., Schürch A.C. (2017). On the (im)possibility of reconstructing plasmids from whole-genome short-read sequencing data. Microb Genomics.

[52] Boolchandani M., D’Souza A.W., Dantas G. (2019). Sequencing-based methods and resources to study antimicrobial resistance. Nat Rev Genet.

[53] Woolhouse M., Ward M., van Bunnik B., Farrar J. (2015). Antimicrobial resistance in humans, livestock and the wider environment. Philos Trans R Soc B-Biol Sci.

[54] Ward M.J., Gibbons C.L., McAdam P.R., van Bunnik B.A.D., Girvan E.K., Edwards G.F. (2014). Time-scaled evolutionary analysis of the transmission and antibiotic resistance dynamics of *Staphylococcus aureus* Clonal Complex 398. Appl Environ Microbiol.

[55] Bloomfield S., Vaughan T., Benschop J., Marshall J., Hayman D., Biggs P. (2019). Investigation of the validity of two Bayesian ancestral state reconstruction models for estimating *Salmonella* transmission during outbreaks. PloS One.

[56] Maio N.D., Wu C.-H., O’Reilly K.M., Wilson D. (2015). New routes to phylogeography: a Bayesian structured coalescent approximation. PLoS Genet.

[57] Hall M.D., Woolhouse M.E.J., Rambaut A. (2016). The effects of sampling strategy on the quality of reconstruction of viral population dynamics using Bayesian skyline family coalescent methods: a simulation study. Virus Evol.

[58] Griffiths E., Dooley D., Graham M., Van Domselaar G., Brinkman F.S.L., Hsiao W.W.L. (2017). Context is everything: harmonization of critical food microbiology descriptors and metadata for improved food safety and surveillance. Front Microbiol.

[59] (2017). AMR Framework for Action Supported by the IACG (2017) Interagency Coordination Group on Antimicrobial Resistance (IACG).

[60] Livernash R. (2019). Pulling together to beat superbugs. Knowledge and implementation gaps in addressing antimicrobial resistance.

